# Prone Regions of Zoonotic Cutaneous Leishmaniasis in Southwest of Iran: Combination of Hierarchical Decision Model (AHP) and GIS

**Published:** 2019-09-30

**Authors:** Elham Jahanifard, Ahmad Ali Hanafi-Bojd, Hossein Nasiri, Hamid Reza Matinfar, Zabihollah Charrahy, Mohammad Reza Abai, Mohammad Reza Yaghoobi-Ershadi, Amir Ahmad Akhavan

**Affiliations:** 1 Department of Medical Entomology and Vector Control, School of Public Health, Tehran University of Medical Sciences, Tehran, Iran; 2 Department of Medical Entomology and Vector Control, School of Public Health, Ahvaz Jundishapur University of Medical Sciences, Ahvaz, Iran; 3 Faculty of Geography, University of Tehran, Tehran, Iran; 4 Department of Soil Science, Collage of Agriculture, Lorestan University, Khoramabad, Iran; 5 Open Training Center, School of Geography, Tehran University, Tehran, Iran

**Keywords:** Decision model, Cutaneous leishmaniasis, Risk map, Iran

## Abstract

**Background:**

Cutaneous leishmaniasis due to *Leishmania major* is an important public health problem in the world. Khuzestan Province is one of the main foci of zoonotic cutaneous leishmaniasis (ZCL) in the southwest of Iran. We aimed to predict the spatial distribution of the vector and reservoir(s) of ZCL using decision-making tool and to prepare risk map of the disease using integrative GIS, RS and AHP methods in endemic foci in Shush (plain area) and Khorramshahr (coastal area) counties of Khuzestan Province, southern Iran from Mar 2012 to Jan 2013.

**Methods:**

Thirteen criteria including temperature, relative humidity, rainfall, soil texture, soil organic matter, soil pH, soil moisture, altitude, land cover, land use, underground water depth, distance from river, slope and distance from human dwelling with the highest chance of the presence of the main vector and reservoir of the disease were chosen for this study. Weights of the criteria classes were determined using the Expert choice 11 software. The presence probability maps of the vector and reservoir of the disease were prepared with the combination of AHP method and Arc GIS 9.3.

**Results:**

Based on the maps derived from the AHP model, in Khorramshahr study area, the highest probability of ZCL is predicted in Gharb Karoon rural district. The presence probability of ZCL was high in Hossein Abad and Benmoala rural districts in the northeast of Shush.

**Conclusion:**

Prediction maps of ZCL distribution pattern provide valuable information which can guide policy makers and health authorities to be precise in making appropriate decisions before occurrence of a possible disease outbreak.

## Introduction

Cutaneous leishmaniasis (CL), a neglected vector-borne disease, is an important public health problem in various parts of Iran. Two endemic forms of CL have been identified in the country: zoonotic cutaneous leishmaniasis (ZCL) due to *Leishmania major* and anthroponotic cutaneous leishmaniasis (ACL) due to *L. tropica* (1, 2). Iran is a high-risk country for leishmaniasis with approximately 20,000 reported cases annually, out of which about 80% are ZCL cases (3). Khuzestan Province is one of the main foci of ZCL in the southwest of Iran (4). In 2012, annual incidence of cutaneous leishmaniasis was 9.5 to 21.7 per 100000 inhabitants in the province (5). This variation may be due to migration of non-immune persons to endemic areas, living people near rodent colonies, migration of rodent and increasing synanthropic index for the reservoir and also lack of knowledge and attitude toward zoonotic cutaneous leishmaniasis (6).

Comprehensive epidemiological and molecular studies in Iran have indicated that *Phlebotomus* (*Phlebotomus*) *papatasi* is the main vector of the disease (2, 7–9). The presence of this sand fly has been reported in different parts of Khuzestan Province (10–13). A high synanthropic index in this species in their natural habitats in Shush and Khorramshahr counties is reported (14). Natural promastigote infection of *Ph. papatasi* has been reported in Khuzestan (12). Moreover, *L. major* has been detected and isolated from this species in Roffyeh district of the province (15).

Based on the distribution of rodent reservoirs of the zoonotic disease, 4 foci of ZCL have been identified in the country. *Leishmania major* was detected in *Tatera indica* (Rodentia: Cricetidae) and *Nesokia indica* (Rodentia: Muridae) using PCR technique in the west and southwest of Khuzestan (16, 17).

Risk factors such as development of agricultural projects, travelling to the endemic areas, road construction, land cover, groundwater level, sand fly and rodent distribution, soil type, humidity, composting animal manures around homes, presence of secondary reservoirs such as dogs, socio-economic status, sleeping outside, precipitation, altitude, temperature, and the species composition of sand flies have all contributed to the increase in the prevalence of zoonotic cutaneous leishmaniasis (4, 18, 19).

Cross-sectional studies on the identification and detection of the *Leishmania* parasite in sand flies showed the distribution of the vector and the disease in different geographical areas of the world, however, the incidence of the disease could not be predicted (20). Geo-environmental factors, which play important role in the spread of ZCL, are often neglected (21). Passive and active surveillances of the disease can be useful for finding and monitoring patients in endemic areas, particularly in the in itial stages of an outbreak (22).

The spatial distribution of the main vector of ZCL is mainly affected by relative humidity and temperature (23). Leishmaniasis occurrence is correlated with environmental variables such as the El Nino phenomena, which is the effect of climate change on sand fly and reservoir population, parasites and emergence and transmission of leishmaniasis (24). Based on the effects of environmental factors on cutaneous leishmaniasis, risk maps of the disease have been prepared in various regions of Iran (21, 25).

Remote sensing (RS) and Geographical Information Systems (GIS) are computer based programs used for determining the presence and abundance of vectors and predicting risk map of vector-borne diseases over the last 25 years (26). RS technique provided outstanding results when used to evaluate the risk of arthropod-borne diseases such as malaria, rift valley fever, West Nile, Lyme, rocky mountain spotted, leishmaniasis and onchocerciasis. Satellite images may be used to determine environmental variables such as land use/land cover and temperature where data are otherwise not available (22).

GIS combines software and hardware systems to analyze, manage and display all spatial distributions in a map framework. GIS technique has been used in some studies for the preparation of risk map of diseases and displaying hazardous zones (26–28). The GIS was used for health policy purposes due to the potential of estimating hospital requirement and health facilities (29). Moreover, GIS is able to determine the correlation between some factors like land use, climatic variables, distance to health center, and malaria transmission (30). This technique has also been used for the study of biodiversity, the presence, and abundance of vector and vector-borne diseases with respect to time (26). Today, it plays an important role in the health sciences, and it is useful in understanding and visualizing disease distribution and epidemiological data (27). Application of GIS in spatiotemporal (space and time) epidemiology research is useful in predicting disease distribution in regions at risk (31, 32). Multiple linear regressions with GIS were used to predict malaria susceptibility zone (33).

Analytic hierarchy process (AHP) technique has been applied in conjunction with GIS and RS to determine at-risk regions in healthcare over the past years (34–36). GIS-based multicriteria decision analysis (MCDA) is the combination of geographical data and valuable judgments of experts that concentrates on solving spatial decision problems (37).

The aims of this study were to determine the spatial distribution of vector(s) and reservoir(s) of ZCL using decision-making tools and to prepare risk map of the disease using integrative GIS, RS and AHP methods in two foci in Khuzestan Province (Shush and Khorramshahr counties). The results of this study can be helpful to health authorities in making precise decisions before disease outbreaks.

## Materials and Methods

### Study areas

**Fig. 1.**
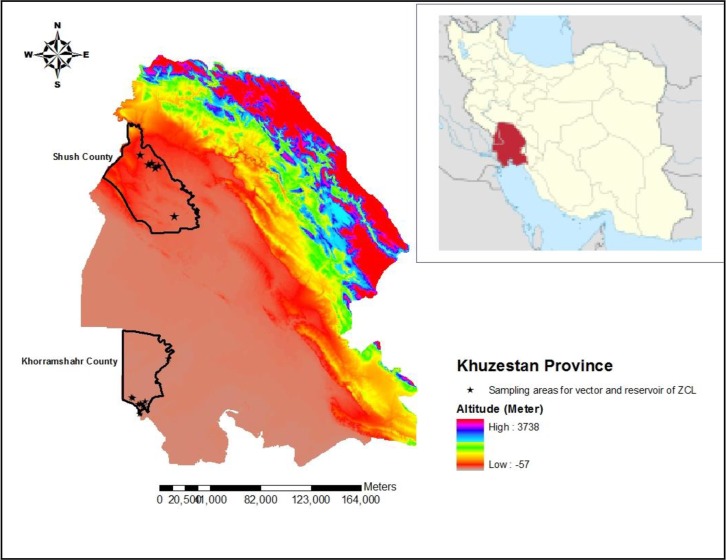
Map of study areas in Khuzestan Province, southern Iran

Khuzestan Province (29° 57′ and 33° 00′ N, 47° 40′ and 50° 33′ E) is one of the 31 provinces in Iran. It is located in the southwest of the country with 27 counties, 76 cities, and 67 rural districts. This province shares border with Iraq and the Persian Gulf. The weather condition is generally warm, but some parts of the northeast have a temperate climate. Three climate zones are identified in the province including mountain, desert, and semi-desert zones. Shush County (32° 11′ 21″ N, 48° 15′ 28″ E) has a land area of 3577km^2^ and it is situated in the northwest of Khuzestan Province with hot and arid weather. The average maximum and minimum temperatures are 46.9 °C and 9.5 °C, respectively. The average annual rainfall in this county is about 180mm. Khorramshahr County (30° 26′ 21″N, 48° 10′ 45″E) is located in the southwest of the province and shares border with Iraq and the Persian Gulf. It is situated at a height of 3 meters above sea level. The weather is hot in summer but mild in winter. The mean maximum and minimum temperatures were 47.5 °C and 9 °C respectively, and mean annual precipitation of 150.6mm in 2013 (Fig. 1). However, Khorramshahr and Shush Counties were regarded as plain and coastal areas in these endemic study areas. Sampling sites were chosen based on villages had the most cases of cutaneous leishmaniasis in the last five years.

### Data collection and preparation

#### Reservoir sampling

The sampling of reservoirs was carried out during four seasons from Mar 2012 to Jan 2013. Rodents were captured mostly during the months of May, July, Sep, Dec, Jan and Feb. Thirty Sherman traps with cucumber, nut, date, bread and butter, Puff and tomato baits were set in natural, agricultural, semi urban and urban ecotypes. Animals were preserved by taxidermy technique and were identified based on morphological characteristics (38). Locations of each of the sampling sites were obtained using GPS technique (Global Positioning System). Only *T. indica* and *N. indica* were included as layers in the Arc Map. These data were used for determining the accuracy of the proposed reservoir map.

### Vector sampling

Vector sampling carried out monthly from 2012 to 2013. Sand flies were collected by 90 sticky traps (30 in human dwelling, 30 in stables and 30 in rodent burrows or outdoor) from various ecotypes (Natural, agricultural, semi-urban and urban) in Shush and Khorramshahr. Trapping was conducted before sunset till sunrise in the next morning and location coordinates were determined using GPS technique. Captured sand flies were preserved in 70% ethanol. Permanent microscopy slides of sand flies were prepared using Puri’s medium (39). All samples were identified using reliable keys (40, 41). Only the collection points of the main vector (*Ph. papatasi*) were included in ArcMap as a layer to determine the accuracy of the model used for the estimation of presence probability of the vector.

### Human infection and eco-environmental data

Data on cases of human leishmaniasis due to *L. major* infection were collected from the Health Centers at Khorramshahr and Shush counties from 2004 to 2011. Indian Remote Sensing (IRS) satellite images, topographical, land use, river and soil maps, and point shape-file of the rural areas were obtained from the Iranian National Geographical Organization of Armed Forces. Distance from human dwelling and river layers were estimated using the buffer operation in ArcGIS. Climate variables were collected from the Meteorological Organization of Khuzestan Province. The annual averages of precipitation, humidity and temperature raster layers were obtained by IDW (Inverse Distance Weighted model) surface analysis of the data. These layers were then clipped based on the boundaries of the study area (Shush and Khorramshahr counties). Water information was obtained from the Water Research Center of Iran. Databank in excel format included water and disease cases used as layers in ArcGIS 10.2 software.

ENVI (Environment for Visualizing Images) software was employed for analyzing the IRS images. Normalized difference vegetation index (NDVI) was calculated and used as land cover map in the modeling process.

### AHP model and processing

The analytic hierarchy process (AHP) is a decision-making tool based on mathematics, pioneered by Thomas L. Saaty in the1970s (42). It has been applied in several decision-making scenarios such as choosing the best alternative, prioritizing or determining the relative merit among a set of alternative, resource allocation, bench markings and quality management (43).

This Multi-Criteria Decision Making (MCDM) technique consists of an ultimate goal, series of alternatives for reaching the goal and a group of criteria that evaluates the alternatives for reaching the goal. It helps decision makers to evaluate factors by pairwise comparisons using standard scales (Table 1) (44). The numerical scale (1–9) reflects the importance of one factor relative to others. Comparison with the numerical scale also shows which elements are more dominant over the others.

**Table 1.** Fundamental scale to pairwise comparison (44)

**Intensity of importance**	**Definition and explanation**
**1**	Equal importance (two activities contribute to the objective)
**3**	Moderate importance(experience and judgment slightly favor one activity over another)
**5**	Strong importance (Experience and judgment strongly favor one activity over another)
**7**	Very strong importance (an activity is favored very strongly over another)
**9**	Extreme importance (the evidence favoring one activity over another is of the highest possible order of affirmation)
**2, 4, 6, 8**	Intermediate values between the two adjacent judgments (when comparison is needed)

Analytic hierarchy process can be summarized in three main steps:

- Arranging the elements in a hierarchy such that the goal of the decision making occupies the top level, with criteria and alternatives occupying intermediate and lower levels, respectively.
- Establishing the matrices of pairwise comparison (judgment matrices) and determining priorities among the elements.
- Checking judgment consistency assigned by the consistency ratio (CR). Saaty (42) suggested that CR less than 0.1 is acceptable whilst CR more than 0.1 needs judgment revision.

Thirteen criteria including temperature, relative humidity, rainfall, soil texture, soil organic matter, soil pH, soil moisture, altitude, land cover, land use, distance from human dwellings, underground water depth, and distance from rivers with a higher probability of presence and abundance of the main vector (*Ph. papatasi*) of ZCL were chosen, but slope element was used instead of distance from human dwellings for the study conducted on the presence and abundance of the main reservoirs (*T. indica* and *N. indica*) in the study areas.

Pairwise comparison matrix was designed based on thirteen criteria by Iranian leishmaniasis experts who have expertise in the ecology and biology of ZCL vector and reservoirs; climatic and environmental factors affecting in the spatial distribution of *Ph. papatasi*, *T. indica* and *N. indica*. The experts’ answers were based on Saaty’s pairwise comparison method (Table 1). The weights of factors affecting the spatial distribution of vector and reservoirs of ZCL were determined by Expert Choice 11 software. The judgment matrixes were constructed regarding sub-criterion (divisions of criteria) and completed using fundamental scale to pairwise comparison by Iranian leishmaniasis experts. Then, the matrix tables were analyzed by Expert Choice 11 software. Every layer was reclassified according to sub-criteria matrix analysis. Final maps were obtained by multiplying standard weight derived from experts’ ideas by weighted maps of the criteria. Hazard map of cutaneous leishmaniasis due to *L. major* was obtained by overlaying the probability maps of vector and reservoirs presence in the study areas.

The presence probability maps of vector and reservoirs e were divided into five classes using the natural breaks method in ArcMap (very low, low, moderate, high and very high), and only areas with high and very high risk were mapped as hazard zones. The high and very high strata were considered as the hot spots for ZCL transmission.

### Accuracy assessment

The accuracy of the presence probability maps of the vector and reservoir were calculated by overlaying the shape files of *Ph. papatasi*, *T. indica* and *N. indica* to the proposed maps in Arc Map. Only vector and reservoir sampling sites with high and very high classes, according to the prepared maps, were considered and the results were reported as a percentage.

## Results

Comparison matrices of the thirteen criteria affecting the presence probability of the vector and reservoirs in Shush and Khorramshahr counties are presented in Tables 2 and 3. The weight of each criterion is showed in Table 4. CR was less than 0.1 in all pairwise comparison matrices. The experts’ opinion indicated that the most effective factor affecting the spatial distribution of the ZCL vector is temperature followed by humidity and precipitation. On the other hand, the most important factor affecting the presence of the reservoir was soil texture followed by land cover and land use.

**Table 2.** Comparison matrix of the criteria affecting the presence probability of the main vector (*Phlebotomus papatasi*)

**Criteria**	**Temperature**	**Relative humidity**	**Rainfall**	**Soil texture**	**Soil organic matter**	**Soil pH**	**Soil moisture**	**Altitude**	**Land use**	**Land cover**	**Distance from human dwelling**	**Underground water depth**	**Distance from river**
**Temperature**	1	2.605	3.728	5.194	5.194	4.384	2.702	5.674	4.02	2.477	1.431	3.347	3.126
**Relative humidity**	-	1	2.724	2.569	3.987	4.555	2.569	4.241	3.758	2.239	1.037	3.936	4.02
**Rainfall**	-	-	1	3.728	3.987	4.416	1.393	4.241	1.644	1.246	0.591	3.882	3.707
**Soil texture**	-	-	-	1	2.537	3.017	0.903	4.004	1.719	1.673	0.684	3.022	3.898
**Soil organic matter**	-	-	-	-	1	2.993	1.059	3.005	1.719	1.149	0.591	2.237	3.594
**Soil pH**	-	-	-	-	-	1	0.506	1.516	0.422	0.384	0.476	0.974	1.105
**Soil moisture**	-	-	-	-	-	-	1	3.692	2.016	2.477	0.728	3.63	3.845
**Altitude**	-	-	-	-	-	-	-	1	0.833	1.099	0.459	2.112	2.091
**Land use**	-	-	-	-	-	-	-	-	1	1.262	0.59	2.605	2.713
**Land cover**	-	-	-	-	-	-	-	-	-	1	2	2.569	3.594
**Distance from human dwelling**	-	-	-	-	-	-	-	-	-	-	1	2.085	2.876
**Underground depth water**	-	-	-	-	-	-	-	-	-	-	-	1	0.994
**Distance from river**	-	-	-	-	-	-	-	-	-	-	-	-	1

**Table 3.** Comparison matrix of the criteria affecting the presence probability of main the reservoirs (*Tatera indica* and *Nesokia indica*)

**Criteria**	**Temperature**	**Relative humidity**	**Rainfall**	**Soil texture**	**Soil moisture**	**Soil organic matter**	**Soil pH**	**Slope**	**Altitude**	**Land cover**	**Land use**	**Underground water depth**	**Distance from river**
**Temperature**	1	2.569	3.201	0.762	1.695	2.424	2.268	1.516	3.245	0.718	0.693	2.713	0.944
**Relative humidity**		1	1.585	0.703	0.725	1.461	1.398	1.431	1.741	0.578	0.509	1.719	0.616
**Rainfall**	-	-	1	0.520	1.059	0.922	1.563	1.246	1.888	0.328	0.509	1.046	0.631
**Soil texture**	-	-	-	1	3.273	3.277	4.258	5.357	5.123	1.431	2.667	4.324	2.954
**Soil moisture**	-	-	-	-	1	1.974	1.585	3.438	3.227	0.544	0.474	1.572	0.668
**Soil organic matter**	-	-	-	-	-	1	1.657	2.512	2.268	0.564	0.552	1.552	0.725
**Soil pH**	-	-	-	-	-	-	1	0.833	1.217	0.343	0.328	1	0.484
**Slope**	-	-	-	-	-	-	-	1	1.38	0.333	0.340	1.585	0.525
**Altitude**	-	-	-	-	-	-	-	-	1	0.476	0.527	1.32	0.668
**Land cover**	-	-	-	-	-	-	-	-	-	1	2.29	4.891	3.129
**Land use**	-	-	-	-	-	-	-	-	-		1	5.619	3.064
**Underground water depth**	-	-	-	-	-	-	-	-	-	-	-	1	0.53
**Distance from river**	-	-	-	-	-	-	-	-	-	-	-	-	1

**Table 4.** The weight of each criterion affecting the presence of reservoirs and vector derived from AHP technique

**Criteria**	**Temperature**	**Relative humidity**	**Rainfall**	**Soil texture**	**Soil organic matter**	**Soil pH**	**Soil moisture**	**Altitude**	**Land use**	**Land cover**	**Distance from human dwelling**	**Underground water depth**	**Distance from river**	**Slope**	**Total**

**AHP Weight**															
**Vector**	0.207	0.148	0.11	0.077	0.06	0.03	0.08	0.034	0.051	0.055	0.095	0.027	0.026	-	1
**Reservoir**	0.105	0.063	0.052	0.174	0.058	0.038	0.072	0.036	0.117	0.139	-	0.034	0.072	0.04	1

**Fig. 2.**
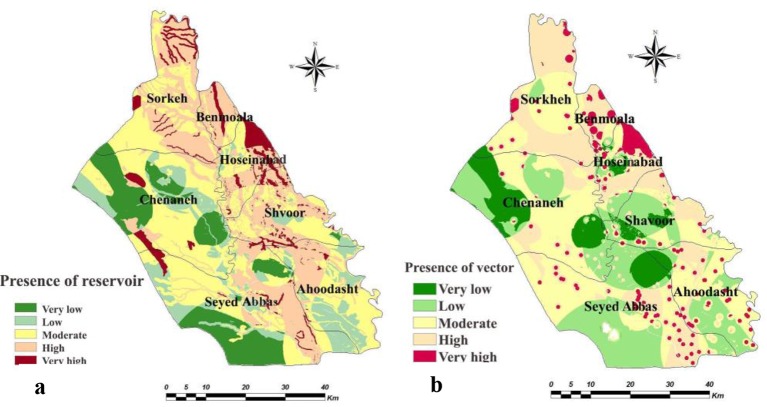
Presence probability maps of (a) reservoir and (b) vector in Shush County, Khuzestan Province of Iran, 2014

**Fig. 3.**
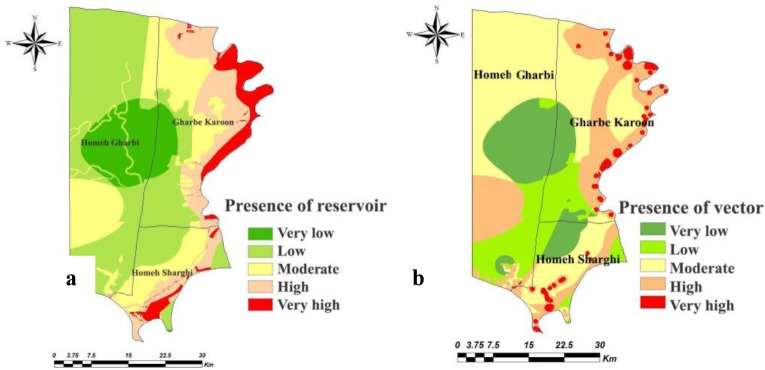
Presence probability maps of (a) reservoir and (b) vector in Khorramshahr County, Khuzestan Province of Iran, 2014

**Fig. 4.**
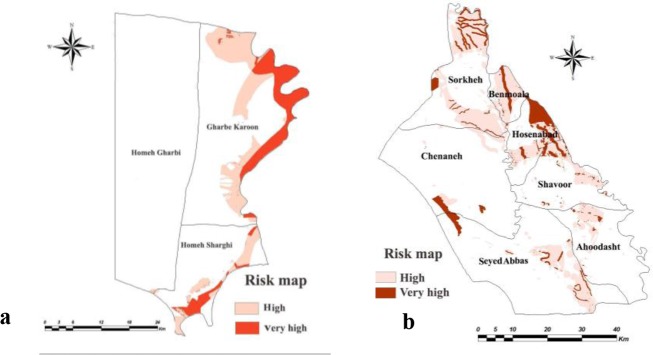
Risk map of zoonotic cutaneous leishmaniasis in (a) Khorramshahr and (b) Shush Counties, Khuzestan Province of Iran, 2014

The weighted maps of layers like temperature, relative humidity, rainfall, soil texture, soil organic matter, soil pH, soil moisture, altitude, land cover, land use, distance from human dwelling, underground water depth, distance from river and slope were prepared for both counties. The presence probability maps of the vector and reservoirs of each county were calculated in ArcMap by multiplying weight of the criteria in their weighted maps (Figs. 2 and 3). Through the combination of the presence probability maps for *Ph. papatasi*, *N. indica* and *T. indica*, the ZCL risk maps in Shush and Khorramshahr Counties were derived (Fig. 4).

Based on the maps derived from the AHP model, in Khorramshahr study area, the highest probability of vector existence is predicted in Gharbe Karoon and small part of Homeh Gharbi rural districts and the highest probability of reservoir presence and ZCL risk was found in Gharbe Karoon rural district. There was no risk of disease transmission in Homeh-Gharbi rural district.

The presence probability of the vector was high in Sorkheh rural district in the north of Shush, but the highest probability of the existence of this species was found in Hossein Abad and Benmoala rural districts in the northeast of Shush County. The presence probability maps of the reservoirs showed that the probability of reservoirs presence was high in Benmoala. However, the risk of ZCL was found to be higher in Hossein Abad and Benmoala rural districts.

**Fig. 5.**
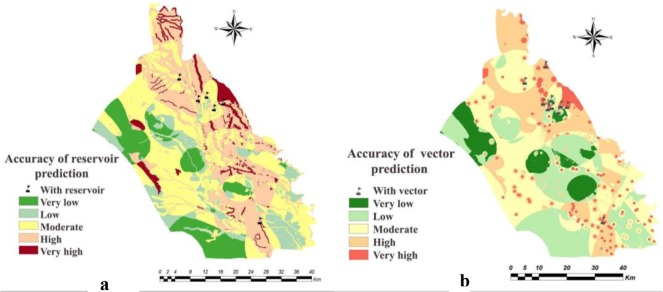
Accuracy of distribution probability maps of (a) reservoir and (b) vector based on analytic hierarchy process model in Shush County, Khuzestan Province of Iran, 2014

**Fig. 6.**
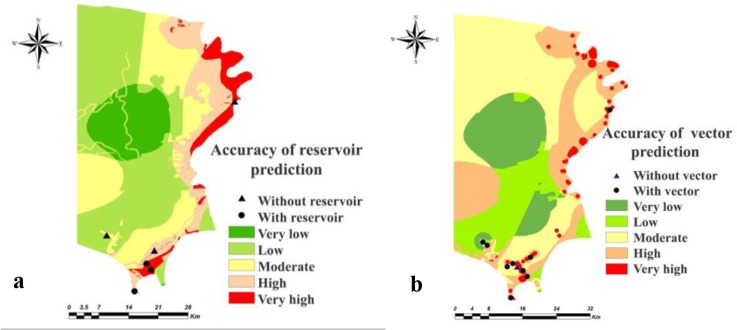
Accuracy of distribution probability maps of (a) reservoir and (b) vector based on analytic hierarchy process model in Khorramshahr County, Khuzestan Province of Iran, 2014

The accuracy of the models used for the estimation of presence probability of the vector and reservoirs were 90% and 75% respectively in the study areas of Shush (Fig. 5) and 80% and 83.33%, respectively, in Khorramshahr County (Fig. 6).

Comparison between the ZCL case distribution and spatial distribution of the vector (*Ph. papatasi*) and reservoirs (*T. indica* and *N. indica*) in the two counties showed that majority of the ZCL cases were reported from the high and very high strata of the presence probability of vector and reservoirs. In other words, the spread of cutaneous leishmaniasis due to *L. major* was directly related to vector and reservoirs’ distribution probabilities.

## Discussion

This study provides spatial distribution of the main vector and reservoirs and risk map of ZCL using decision-making tools and integrated GIS and RS in Shush and Khorramshahr counties in the southwest of Iran. Data on human leishmaniasis cases collected from the two counties show that most of the ZCL cases occurred in Benmoala, Hossein Abad, Shavoor and Sayedabbas rural districts of Shush and Homeh-Sharghi rural district of Khorramshahr, where there are many agricultural fields. The hazard maps of the disease revealed that most of the cases were recorded in areas where there are agricultural lands. Human factors such as population pressure, urbanization and new agricultural projects affect the distribution of leishmaniasis. Land use including agricultural lands, rivers, canals and irrigation water are suitable circumstances for the growth of vectors and provide nesting habitats for reservoirs (45, 46). Sand flies prefer humid habitats for the laying of eggs, survival, and development of immature stages (47). Inland waters and streams affect the abundance and distribution of vectors. Increase in sand fly abundance can be facilitated by shortening the reproductive cycle due to an increase in surface moisture in areas such as inland waters and river banks. In the central part of Bihar in eastern India, inland water bodies increased the transmission risk of visceral leishmaniasis (VL) by providing suitable breeding places for the vector *Ph. argentipes* (48). In the present research study, disease cases and spatial distribution maps of *Ph. papatasi* derived from a combination of AHP and GIS revealed that most of the cases due to *L. major* occurred in areas with high and very high presence probability of sand flies. A decrease in the depth of underground water provides suitable condition such as moisture, critical factor that increases the population of sand flies.

Sand fly activities are strongly associated with two environmental parameters; precipitation and temperature (49). Precipitation, mean temperature and slope are the main environmental variables that affect the distribution of the main ZCL vector in Iran, using the MaxEnt model (50). Increased vector abundance is due to the presence of infected rodent and increased soil moisture in the region. The epidemiology of CL is mostly related to the spatiotemporal distribution of the vector and reservoir (49). There is a correlation between the geographical distribution of zoonotic cutaneous leishmaniasis and its main vector, and *L. major* has been frequently isolated from *Ph. papatasi* in various foci in the country (2).

Several studies have been conducted on the effects of environmental factors on the distribution and incidence of CL (51, 52). The risk map of visceral leishmaniasis was prepared in two districts in East Azarbaijan Province (Ahar and Kalaybar) using group decision-making tools and GIS (34). Fuzzy AHP model is widely used as a predictor of the prevalence of the disease with an accuracy of more than 80%. In a research study, among eight parameters only dogs, nomads and altitude were the most effective factors affecting the incidence of VL. Two studies have been conducted on mapping hazard areas of ZCL in Esfahan and Golestan Provinces using the fuzzy AHP method (21, 25). Altitude and land cover have a negative influence on disease incidence. Temperature and relative humidity are the two main factors that directly correlate with the incidence of ZCL (21). Environmental factors that affect vector and reservoir population influence the spread and incidence of leishmaniasis. Moreover, distribution of cutaneous and visceral leishmaniasis was found to be affected by temperature fluctuations (53).

In the present research, variables such as soil texture, land cover, land use and temperature, in order of descending influence, were found to have the highest impact on the presence probability of the reservoirs. The most effective criteria affecting the presence probability of *Ph. papatasi,* based on experts’ judgment, were temperature, relative humidity and precipitation. There were some differences between our results and that of some previous studies on leishmaniasis. The difference may be due to the different forms of the disease studied and the various criteria selected for the study. In some previous study, criteria such as average altitude, health center distance, population and evaporation were chosen whilst ignoring the two most important factors of ZCL cycle like vector and reservoir in the preparation of hazard maps of the disease. Environmental elements like slope and seasonal precipitation contribute to the spreading prediction of *N. indica* and *T. indica* (ZCL reservoirs), respectively, in Iran (17).

The risk map of Kala-azar was prepared using geo-environmental factors such as land cover/land use, condition of vegetation, surface dampness, climate, illiteracy and unemployment rate of the inhabitants in the Vaishali District of Bihar in India (48).

In the present investigation, the hazard map of ZCL was prepared using climate and environmental elements in Shush and Khorramshahr counties. According to the multi-criteria decision-making technique, the highest probability of the disease presence is predicted in Gharb-e- Karoon rural district of Khorramshahr whilst Sorkheh, Hoseinabad and Benmoala rural districts where the high-risk areas of ZCL in Shush County. Different multi-criteria decision analysis techniques for disease susceptibility mapping were compared (54). AHP model, with high accuracy, was the best among the tools used for the prediction of VL in the northwest of Iran and dengue virus disease in Ecuador.

## Conclusion

Using a combination of GIS, RS and AHP decision-making techniques, valuable risk maps of cutaneous leishmaniasis due to *L. major* were prepared while GIS and RS are judged only on the base of evidence and environmental factors, and there is no possibility of intervention by experts. Our study confirms the usefulness of AHP and GIS techniques in preparing the spatial distribution of *Ph. papatasi*, *N. indica* and, *T. indica* and the risk map of ZCL in areas where similar vector and reservoirs exist. Furthermore, it is possible to update the maps by entering new data and satellite images.

In the present study, eco-environmental factors were found to have a greater influence on the presence of vector and reservoirs, which can affect the incidence of ZCL. We recommend the use of Multi-Criteria Decision Making technique for the preparation of risk maps of ZCL in other counties of the province. The proposed maps are visual tools that highlight areas with the potential for the disease transmission and/ or outbreak and the population at risk. Educating and increasing the knowledge of the people on reservoir, vector, transmission and control of the disease is deemed necessary. In addition, hazard maps are valuable data for health authorities in allocating budget for effective disease control programs and health care for the people living in high-risk zones.
